# Healthy Lifestyle, Genetic Risk and Brain Health: A Gene-Environment Interaction Study in the UK Biobank

**DOI:** 10.3390/nu14193907

**Published:** 2022-09-21

**Authors:** Anwar Mulugeta, Shreeya S. Navale, Amanda L. Lumsden, David J. Llewellyn, Elina Hyppönen

**Affiliations:** 1 Australian Centre for Precision Health, Unit of Clinical and Health Sciences, University of South Australia, Adelaide, SA 5001, Australia; 2 South Australian Health and Medical Research Institute, Adelaide, SA 5000, Australia; 3 Department of Pharmacology and Clinical Pharmacy, College of Health Science, Addis Ababa University, Addis Ababa P.O. Box 9086, Ethiopia; 4 College of Medicine and Health, University of Exeter, Devon EX1 2LU, UK; 5 Alan Turing Institute, London NW1 2DB, UK

**Keywords:** healthy lifestyle, smoking, obesity, physical activity, healthy diet, genetic risk, magnetic resonance imaging, UK Biobank

## Abstract

Genetic susceptibility and lifestyle affect the risk of dementia but there is little direct evidence for their associations with preclinical changes in brain structure. We investigated the association of genetic dementia risk and healthy lifestyle with brain morphometry, and whether effects from elevated genetic risk are modified by lifestyle changes. We used prospective data from up to 25,894 UK Biobank participants (median follow-up of 8.8 years), and defined healthy lifestyle according to American Heart Association criteria as BMI < 30, no smoking, healthy diet and regular physical activity). Higher genetic risk was associated with lower hippocampal volume (beta −0.16 cm^3^, 95% CI −0.22, −0.11) and total brain volume (−4.34 cm^3^, 95% CI −7.68, −1.01) in participants aged ≥60 years but not <60 years. Healthy lifestyle was associated with higher total brain, grey matter and hippocampal volumes, and lower volume of white matter hyperintensities, with no effect modification by age or genetic risk. In conclusion, adverse effects of high genetic risk on brain health were only found in older participants, while adhering to healthy lifestyle recommendations is beneficial regardless of age or genetic risk.

## 1. Introduction

Globally, 55.2 million people are living with dementia, with the overall prevalence rising due to an aging population [1]. Preclinical changes to the brain begin long before the presentation of dementia symptoms [2], and several aspects of brain morphology are linked to an increased dementia risk [3]. For instance, hippocampus is a region in brain which has a major role in learning and memory, and hippocampal atrophy has been suggested as early marker of dementia and cognitive decline [3,4]. Another often used marker is white matter hyperintensity volume, which is an indicator of lesions in the white matter of the brain that are often seen in the dementia subtypes of Alzheimer’s disease (AD) and vascular dementia [5]. Reductions in grey and white matter volumes have been reported across multiple disease sub-types [6,7], highlighting the great interest in understanding drivers of brain atrophy. Indeed, as efforts to prevent dementia are the most likely to be effective when implemented at the early stages of the pathogenic processes, understanding how differences in lifestyle factors affect structural changes to the brain may help in revising effective strategies for prevention.

There are encouraging data to suggest that preventing, or at least, delaying the onset of dementia is possible, and lifestyle factors such as maintaining a healthy weight, not smoking, undertaking physical activity, and consuming a healthy diet may all reduce the risk of AD and dementia [8,9]. Less is known about the associations between lifestyle differences and brain morphology, and while some studies have investigated associations for individual factors such as physical activity or diet [10,11], their effects have not been investigated in combination in longitudinal studies. However, there are promising data from a 2-year lifestyle intervention study, suggesting improved cognitive performance in the group randomized to the ‘active group’ (n = 631) compared to placebo (n = 629) [12] although a follow up sub-study with complete brain imaging outcomes (n = 132) did not find significant differences in brain morphology between the two groups [13]. In observational studies, normal weight [14], not smoking [15,16] and taking part in regular physical activity [10] have all been individually associated with favorable brain outcomes, including higher total brain, grey matter, and hippocampal volumes, and less white matter hyperintensity lesions. Another important component of lifestyle is diet, and while mounting evidence supports beneficial associations between an overall healthy diet and brain health [11], for individual dietary components the results are sometimes contradictory [11,17,18]. Importantly, many of the lifestyle–brain volume associations have been studied in people aged >60 years and since the development of dementia may start decades earlier [2], further work is needed to identify drivers of early changes in brain morphology.

In this study, we examine the prospective associations of healthy lifestyle score and its components with measures relating to brain health using data from up to 25,894 middle to older age participants from the world’s largest neuroimaging study. Furthermore, since dementia risk is at least in part conferred by genetic factors [19], we look at the associations between genetic AD risk and preclinical alterations in brain morphology, and whether by adhering to healthy lifestyle recommendations, it may be possible to mitigate these risks.

## 2. Materials and Methods

### 2.1. Study Population

The UK Biobank is a prospective cohort of over 500,000 individuals aged 37–73 years at the time of recruitment between 2006 and 2010 [20]. Extensive data were obtained from questionnaires, interviews, physical measures, and biological samples, and through linkage with health records and death registries [20]. An imaging sub-study was initiated in 2014 and information on neuroimaging outcomes have been made available for over 45,000 participants as of early 2020 [21]. Our analyses were restricted to unrelated white British individuals with complete genetic, lifestyle and socioeconomic information and no dementia at baseline based on the information available from self-report, hospital admission health records, primary care data, and death registry (Figure S1). Participants with no neuroimaging data (n = ~283,000) and those with outlier brain volume data (~600) were excluded, leaving up to 25,894 individuals (up to 8302 age 60 and above) for the analyses. Ethical approval for the UK Biobank was granted by the National Information Governance Board for Health and Social Care and North West Multicenter Research Ethics Committee (11/NW/0382). All participants provided electronic consent to use their anonymized data including their linked registry-based records for health-related research [20]. This study was conducted under UK Biobank application number 10171.

### 2.2. Healthy Lifestyle Factors

We defined the healthy lifestyle score based on established healthy lifestyle factors reported in the American Heart Association (AHA) guidelines [22]. Having a body mass index (BMI) below 30, being currently a non-smoker, undertaking regular physical activity, and having a healthy diet, each of which scored 1, were used to construct the healthy lifestyle score, with the combined value ranging from zero (least healthy) to four (most healthy). A ‘healthy diet’ was defined by a healthy diet score adapted from the AHA guidelines [22] and included the following three components: (1) fruit and vegetable intake: >4.5 pieces or serving intake per day (code 1 otherwise 0); (2) total fish intake: two or more times per week (code 1 otherwise 0); and (3) low meat intake: two or fewer times per week processed meat and five or fewer times per week red meat (code 1 otherwise 0). Abidance by at least two of the healthy items was considered to define a “healthy diet”. In relation to physical activity, the AHA guidelines recommend at least 150 min of moderate-intensity or 75 min of vigorous-intensity physical activity per week or an equivalent combination [22], and we defined regular activity accordingly (Table S1). Information on these lifestyle factors was based on self-reported data from the touchscreen questionnaire administered during the baseline survey at recruitment, and prior to using the healthy lifestyle score and its components for the main analysis, we checked whether they were associated with mortality risk in the expected directions (Text S1 and Figure S2). Information on questions used for relevant lifestyle indicators, demographic and socioeconomic covariates is provided in Table S1. We also used ‘24 h dietary recall’ data available for a subsample of UK Biobank participants to check their association with the respective ‘food frequency’ data (available for all) that we used in generating the healthy diet (Table S2).

### 2.3. Neuroimaging Outcomes

Brain magnetic resonance imaging (MRI) data were collected for a subsample of UK Biobank participants at four study sites (Stockport (Cheadle), Reading, Newcastle, and Bristol), and raw imaging data were further processed by the UK Biobank to provide image-derived phenotypes for researchers’ use [23]. Details on the imaging procedures, processing pipeline and derivation of imaging-derived phenotypes have been discussed in prior studies [23,24]. Briefly, brain imaging was carried out using a Siemens Skyra 3T scanner (running on VD13A SP4 software) with a standard Siemens 32-channel RF receive head coil and with the scan covering from the top of the cranium to the neck/mouth region [23]. T1-weighted and T2-FLAIR structural imaging were acquired using straight sagittal orientation and were centrally preprocessed to derive brain volumetric measures including total brain volume, grey matter volume, white matter volume, hippocampal volume, and volume of white matter hyperintensities, which we used in this study. The brain volumes were calculated using FreeSurfer software, and we normalized for participant head size using a T1-based head sizing scaling factor (scaled brain volume = brain volume × head size scaling factor) [23], and log-transformed the volume of white matter hyperintensities for approximating normal distribution.

### 2.4. Genetic Risk Score

Genetic data were extracted from the third release UK Biobank genome-wide data, and details on genotyping and imputation procedures and related quality control measures have been reported elsewhere [25]. In brief, genotyping was performed using the UK BiLEVE array (n~50,000) and UK Biobank axiom array (~438,000), with the two arrays having around 95% marker similarity. Imputation was carried out using the Haplotype Reference Consortium, UK10K and 100 genome Phase 3 reference panels [25]. For the current analysis, we used 22 AD risk-increasing genetic variants identified in the latest meta-analysis of genome-wide association studies (GWAS) involving over 28,000 AD cases and over 74,000 controls, with no contribution from the UK Biobank [19] (see the criteria for GWAS selection in Text S2; and variants used are listed in Table S3, imputation quality score ≥ 0.96 and minor allele frequency ≥ 0.02). We constructed the weighted genetic risk score for each individual as the sum of the number of AD risk-increasing variants weighted by the coefficient (log odds of variant–AD association) taken from the discovery meta-analysis of GWAS (Text S2) [19].

### 2.5. Statistical Analysis

We used linear regression to test the association of the genetic risk score and healthy lifestyle score with neuroimaging outcomes, in three models. In the first model we adjusted for basic covariates including age, sex, assessment center and duration until imaging (in years). In the second model, we further adjusted for socioeconomic factors including education, employment, and Townsend deprivation index. In the final model we further adjusted for alcohol consumption and long-standing illness (covariates used described in Table S1). Analyses involving genetic risk score were additionally adjusted for 40 principal components and genotyping array. Effect modification between the continuous terms of genetic risk score and healthy lifestyle score on brain neuroimaging markers was tested including an interaction term in the linear regression model. Based on the genetic and lifestyle risk, we further categorized the population as “favorable lifestyle and low genetic risk (individuals with healthy lifestyle score of three or four and in 1st and 2nd tertile of genetic risk score)”, “unfavorable lifestyle and low genetic risk (healthy lifestyle score of ≤2, and in the 1st and 2nd tertile of genetic risk)”, “favorable lifestyle and high genetic risk (healthy lifestyle score of three or four, and in the highest tertile of genetic risk)”, and “unfavorable lifestyle and high genetic risk (healthy lifestyle score of ≤2, and in the highest tertile of genetic risk)” groups. Using the favorable healthy lifestyle and low genetic risk group as a reference, we fitted a linear regression against the neuroimaging outcomes to further understand the combined genetic and lifestyle effects on brain health.

Additionally, we investigated the association of components of the lifestyle score (i.e., no current smoking, healthy weight, regular physical activity and healthy diet) with brain volumes in a mutually adjusted model additionally containing the basic and socioeconomic covariates, alcohol consumption, and long-standing illness. Given the likelihood that brain neuroimaging changes advance with age, we tested for interaction by age, and repeated the analyses separately for those aged below 60 years and those aged 60 years and above when evidence for effect modification was observed. We applied a multiple test corrected *p*-value depending on the number of tests included in the primary analysis. Accordingly, a significance level of *p* ≤ 0.01 for genetic analysis (0.05/5 outcomes), and *p* ≤ 0.002 for healthy lifestyle score and its components (0.05/(5 outcomes × 5 exposures)) were used. Effect estimates are reported as beta-coefficients and standard error per 10 risk allele higher genetic risk of AD (here after referred as genetic risk), and per one-unit higher healthy lifestyle score. All analyses were performed using the STATA SE version 16.1 software.

## 3. Results

Among 308,846 white British ancestry participants with complete genetic, lifestyle and socioeconomic information, and no history of dementia at the baseline, 25,894 took part in the brain imaging sub-study (53% were women). The average time interval between the baseline and the imaging sub-study was 8.8 years (SD 1.7). The UK Biobank participants included in the imaging sub-study were younger, more educated, more likely to be employed, less deprived, and more likely to have a healthy lifestyle (less current smoking, of healthy weight and more engaged in regular physical activity), and less likely to have a history of long-standing illness at the baseline compared to the full baseline sample (Table S4). Compared to others, men, those aged 60–73 years, those without qualification, with long-standing illness, and with unfavorable lifestyle including current smoking and obesity had smaller grey matter and hippocampal volumes, and higher burden of white matter hyperintensities (Table 1).

**Figure 1:**
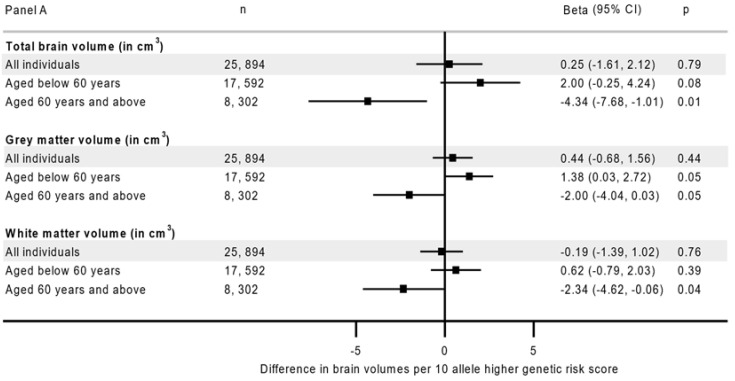

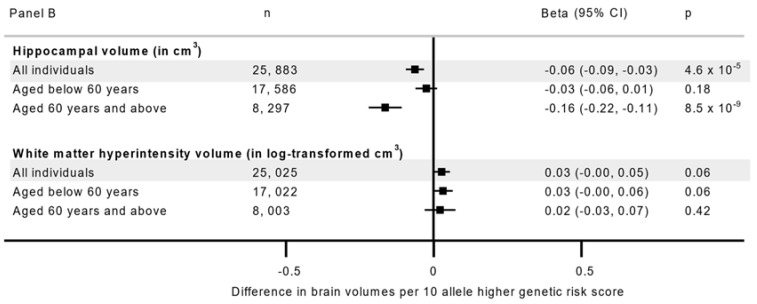
The association between genetic risk and neuroimaging outcomes age-combined and age-stratified analyses. **Panel A**: Total, grey and white matter volumes; **Panel B**: Hippocampal and white matter hyperintensity volumes. Estimates from a model adjusted for age, sex, assessment center, genotyping arrays and 40 principal components. cm^3^ is cubic centimeter.

A higher genetic risk was associated with lower hippocampal volume (beta −0.06 cm^3^, 95% CI −0.09 to −0.03 per 10 allele higher), but not with total brain, grey matter or white matter volumes, or volume of white matter hyperintensities (*p* ≥ 0.06, Figure 1 Panel A & Panel B) in age-combined analyses. However, the associations with total, grey and hippocampal volumes were dependent on age (for all, p_age-interaction_ ≤ 0.007) while no significant variation was observed in white matter volume and white matter hyperintensity volume (for both, p_age-interaction_ ≥ 0.03, Table S5). After age stratification, higher genetic risk was associated with lower hippocampal volume (−0.16 cm^3^, 95% CI −0.22, −0.11, *p* = 8.5 × 10^−9^) in older participants (≥60 years), with no association found in the participants aged <60 years (−0.03 cm^3^, 95% CI −0.06, 0.01, *p* = 0.18, p_age-interaction_ = 6.1 × 10^−5^, Figure 1 Panel B). Higher genetic risk was also associated with lower total brain volume in the older (−4.34 cm^3^, 95% CI −7.68, −1.01, *p* = 0.01) but not in the younger population (2.00 cm^3^, 95% CI −0.25, 4.24, p_age-interaction_ = 0.003), with similar contrast in grey matter volume (Figure 1 Panel A). The genetic association with brain outcomes was unchanged after adjusting for socioeconomic and lifestyle factors (Table S5).

**Figure 2:**
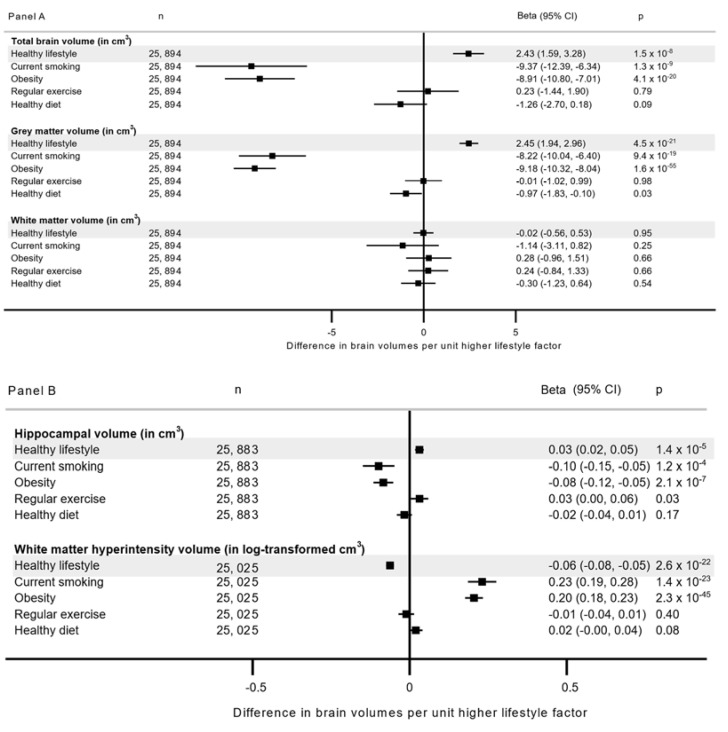
Association between adherence to healthy lifestyle recommendations and neuroimaging outcomes. **Panel A**: Total, grey and white matter volume; **Panel B**: Hippocampal and white matter hyperintensity volume. Estimates are from a model adjusted for age, sex, assessment center, duration until imaging (in years), education, Townsend deprivation index, employment, alcohol consumption, and longstanding illness. The analysis involving individual components of lifestyle score included further adjustment for all other components. cm^3^ is cubic centimeter.

Each unit increase in the healthy lifestyle score was associated with higher total brain volume (2.43 cm^3^, 95% CI 1.59, 3.28 per each additional lifestyle), higher grey matter volume (2.45 cm^3^, 95% CI 1.94, 2.96) and hippocampal volume (0.03 cm^3^, 95% CI 0.02, 0.05) and lower volume of white matter hyperintensities (−0.06 in log-transformed cm^3^, −0.08 to −0.05) in the age-combined analyses (for all, *p* ≤ 1.4 × 10^−5^, Figure 2 Panel A & Panel B), with no significant differences between the younger and older participants (p_age-interaction_ ≥ 0.28, Table S6 and Figure S3).

In our secondary analyses using components of healthy lifestyle, obesity and current smoking were associated with lower total brain, grey matter and hippocampal volumes, and greater burden of white matter hyperintensities (Figure 2 Panel A & Panel B). Some associations of obesity varied by age; obesity was more strongly associated with higher white matter hyperintensity volume in the participants aged <60 years (0.23, 95% CI 0.20, 0.26) than those ≥60 years (0.14, 95%CI 0.09, 0.20; p_age-interaction_ = 2.5 × 10^−4^), while for white matter volume, a positive association was seen in those ≥60 years (3.40, 95% CI 1.09, 5.72) but not in those <60 years (−0.97, 95% CI −2.43, 0.48, p_age-interaction_ = 3.9 × 10^−4^, Table S6 and Figure S3). The AHA recommended level of physical activity or ‘healthy diet’ were not associated with neuroimaging outcomes at multiple testing corrected significant threshold, and we observed no evidence for interaction by age.

We also investigated whether the association between healthy lifestyle and brain morphology varied by genetic AD risk, but we found no evidence of interaction between the genetic risk and healthy lifestyle or its components (p_GRS-interaction_ > 0.02, Table S6). There was no evidence for three-way interaction between age, healthy lifestyle, and genetic risk (Table 2 and Table S7).

## 4. Discussion

Using information from the world’s largest neuroimaging study, we showed an age-specific association between genetic risk of AD with brain health, and confirmed the benefits of adhering to healthy lifestyle recommendations. While any adverse effects of high genetic risk appear to have an effect only at older ages, refraining from smoking and maintaining a healthy weight appeared to be particularly beneficial for brain integrity regardless of age. We found no evidence of a gene-lifestyle interaction, suggesting that lifestyle changes can help support brain health regardless of genetic risk.

To our knowledge, this is the first prospective study to investigate the association between adherence healthy lifestyle recommendations and neuroimaging outcomes. Related effects have been previously investigated in a randomized controlled lifestyle trial in participants at risk of dementia [12,13]. While some evidence of benefits of lifestyle intervention on preserving and maintaining cognitive functioning were observed in the first part of their study (n = 1260) [12], in the small follow-up study including information on neuroimaging outcomes (n = 132) they failed to confirm effects on brain structure [13]. Our analyses included up to 25,894 participants, and we found that a higher healthy lifestyle score was associated with higher total brain, grey matter, and hippocampal volumes, and lower burden of white matter hyperintensities. These associations appeared to be primarily driven by not smoking, and having a healthy weight (BMI < 30 kg/m^2^), consistent with findings from other prospective studies [14,15,16,26]. For example, we have previously provided causal genetic evidence for the association between obesity and lower total brain and grey matter volumes [26]. Evidence of an association with smoking was reported in a recent prospective population-based study (Coronary Artery Risk Development in Young Adults, n = 698 cohort), that detected 8.33 cm^3^ (95% CI −13.44, −4.29) lower grey matter volume among current smokers compared to never-smokers [15], a finding consistent with ours (−8.22, 95% CI −10.04, −6.40) despite our comparison being between current and non-current smokers (never-smokers and ex-smokers). Findings from a meta-analysis suggested that regular physical activity was associated with greater hippocampal volume [10], while no conclusive evidence was found in our study after correcting for multiple comparisons. In line with a previous study using the UK Biobank [27], we did not find robust evidence for an association between diet and brain morphology.

Another important finding of our study is the age-dependent association between genetic risk and neuroimaging outcomes including total brain, grey matter, and hippocampal volumes. Lower hippocampal volume represents a common AD neuroimaging marker, and we found the expected association between higher genetic AD score and lower hippocampal volume, as also supported by earlier studies [28,29,30]. That the adverse genetic association with brain structure is only present in older rather than younger individuals suggests influences operating as part of the active disease development stage, rather than reflecting lifelong differences in brain structure. Indeed, one of the key AD variants, *APOE4,* while increasing the risk in older age disease [31], is also associated with better episodic memory in young and healthy populations [32]. In contrast to the genetic effects, obesity appeared to have a stronger association with the burden of white matter hyperintensities in the younger compared to older participants, although the associations were seen irrespective of age.

Our study did not find evidence of an interaction between healthy lifestyle and genetic AD risk on the association with neuroimaging outcomes, suggesting that adhering to a healthy lifestyle is likely to be beneficial to all individuals irrespective of their genetic risk. In a previous study, the association between a healthy lifestyle and lower risk of incident all-cause dementia was also not modified by genetic risk [33].

Multiple plausible mechanisms may underlie the associations we observed. For example, central adiposity (visceral fat) can contribute to chronic inflammation via the release of pro-inflammatory cytokines, which may be linked to differences in brain morphometry [34]. Inflammatory biomarkers secreted from adipose tissue can cross the blood–brain barrier and further stimulate cytokines production in the brain, disrupting neurocognitive function [35]. We have shown previously that genetically instrumented obesity types that associate with lower grey matter volume (‘metabolically unfavorable’ and ‘neutral adiposity’), are associated with higher levels of inflammation marker C-reactive protein whilst ‘metabolically favorable’ obesity which showed a suggestive association with higher grey matter volume, was not; suggesting inflammation may be related to the grey matter volume-decreasing mechanism [26]. A higher oxidative stress is an another mechanism that may contribute to the observed effects of lifestyles such as smoking on the brain, by causing cellular damage if inadequately neutralized by antioxidants [35,36]. Animal studies have suggested that low amounts of physical activity, exposure to smoking and high amounts of saturated fatty acids can compromise cerebrovascular blood flow and lead to brain atrophy [36,37,38,39]. Additionally, low levels of physical activity and high-fat diets, consumed by obese rats have been associated with lower levels of neurotrophic growth factors and higher levels of inflammatory markers, especially in the hippocampus, and an increase tau-phosphorylation; one of the histopathological hallmarks of AD [36].

Our study has several strengths. To our knowledge, this is the first study to investigate the association between a healthy lifestyle score and its components with neuroimaging outcomes. We used information from a large cohort of middle to older aged adults, with the data allowing for extensive covariate adjustments and exploration of interaction by genetic AD risk. We were also able to provide further insight into age depended influences on brain morphometry, and to investigate these associations with greater power than earlier studies.

Our study also has some limitations which should be considered when interpreting the results. Firstly, the UK Biobank is affected by healthy volunteer bias, whereby the participants are generally healthier, less socioeconomically deprived, more educated and likely to have more favorable lifestyle than the general UK population [40]. Secondly, the information used to assess adherence to healthy lifestyle recommendations were collected at the baseline and this may not capture the effect of previous lifestyle of the participants on brain health. Furthermore, information on smoking, physical activity and diet were self-reported and therefore, may contain some dilution and bias. However, we made extensive efforts to assess the information, including validation of the self-reported food frequency questionnaire against the 24 h recall. We also checked for the association between the self-reported measures reflecting ’healthy lifestyle’ against mortality, confirming the expected associations for all components, except for fish intake that is typically considered part of a healthy diet. However, there are differences between different types of fish, and for example the traditional British dish ‘fish and chips’ consist of white fish that is commonly deep fried and high in saturated fat. Thirdly, despite adjusting for multiple covariates and having a median follow up of 8.8 years, we cannot rule out residual confounding or reverse causation. Finally, our study is restricted to participants of European ancestry, so the findings may not be generalizable to other populations and future studies in more diverse populations are required.

## 5. Conclusions

Genetic AD risk is an important risk factor for total brain and hippocampal volume reductions at an older age, while adherence to healthy lifestyle recommendations appears to be beneficial for brain health regardless of age and genetic AD risk.

## Figures and Tables

**Table 1:** Summary of brain volumes by characteristics of the UK Biobank participants.

Characteristics	N(%)	Grey Matter Volume Median (IQR)	Hippocampal Volume Median (IQR)	White Matter Hyperintensities Median (IQR)
All	25,894	7792.6 (760.7, 825.2)	10.0 (9.2, 10.7)	3.7 (2.0, 7.4)
Sex				
Men	12,286 (47.4)	776.8 (746.8, 806.4) *	9.6 (8.9, 10.3) *	3.8 (2.0, 7.7)
Women	13,608 (52.6)	806.9 (7768., 839.2)	10.2 (9.6, 10.9)	3.5 (1.9, 7.1)
Age				
39–49 years	6839 (26.4)	829.7 (80.2, 855.8)	10.3 (9.7, 10.9)	2.0 (1.2, 3.4)
50–59 years	10,753 (41.5)	794.9 (7689, 821.3)	10.1 (9.4, 10.7)	3.6 (2.1, 6.5)
60–73 years	8302 (32.1)	760.9 (734.2, 786.9) *	9.5 (8.8, 10.2) *	6.5 (3.5, 12.4) *
Education				
None	1535 (5.9)	777.3 (749.1, 807.5) *	9.8 (9.1, 10.5) *	5.5 (2.9, 10.2) *
NVQ/CSE/A-levels	8071 (31.2)	796.2 (762.1, 829.9)	10.0 (9.3, 10.7)	3.6 (2.0, 7.2)
Degree/professional	16,288 (62.9)	792.4 (761.1, 824.2)	10.0 (9.2, 10.7)	3.5 (1.9, 7.1)
Employment				
None	1493 (5.8)	803.1 (772.4, 835.2)	10.1 (9.4, 10.8)	3.3 (1.8, 6.3)
Retired	6580 (25.4)	764.4 (736.8, 792.4) *	9.6 (8.9, 10.3)	6.2 (3.3, 11.9)
Lowest working hour (1st quartile)	4085 (15.8)	801.1 (769.3, 835.3)	10.1 (9.4, 10.8)	3.4 (1.8, 7.0)
2nd quartile	2999 (11.6)	807.6 (775.9, 839.8)	10.2 (9.4, 10.8)	3.0 (1.7, 5.8)
3rd quartile	5732 (22.1)	8804.8 (773.9, 835.5)	10.1 (9.4, 10.8)	2.9 (1.7, 5.4)
Highest working hour (4th quartile)	5005 (19.3)	797.1 (768.6, 826.1)	10.0 (9.3, 10.8)	3.0 (1.7, 5.6)
Townsend deprivation index				
Highly deprived	10,232 (39.5)	791.3 (759.6, 824.0)	10.0 (9.2, 10.7)	3.7 (2.0, 7.5)
Less deprived	15,662 (60.5)	794.3 (761.9, 827.0)	10.0 (9.3, 10.7)	3.6 (1.9, 7.2)
Alcohol				
Non-drinker	1040 (4.0)	795.3 (764.8, 826.3)	10.0 (9.3, 10.7)	3.9 (2.1, 8.4)
Special occasion only	1929 (7.4)	801.0 (770.4, 832.9)	10.1 (9.4, 10.8)	3.7 (2.0, 7.8)
1–3 times/month	2737 (10.6)	805.2 (771.5, 838.1)	10.1 (9.4, 10.8)	3.3 (1.8, 6.7)
1–2 times/week	6682 (25.8)	798.6 (767.6, 830.8)	10.0 (9.3, 10.7)	3.4 (1.8, 6.9)
3–4 times/week	7527 (29.1)	790.8 (760.2, 822.2)	9.9 (9.2, 10.6)	3.6 (1.9, 7.2)
Daily or almost daily	5979 (23.1)	779.0 (747.8, 810.6) *	9.8 (9.1, 10.5) *	4.1 (2.2, 8.1)
Long standing illness				
No	19,920 (76.9)	795.0 (763.0, 827.2)	10.0 (9.3, 10.7)	3.5 (1.9, 6.9)
Yes	5974 (23.1)	784.3 (753.0, 817.6) *	9.8 (9.1, 10.6) *	4.3 (2.2, 8.8) *
Current smoking				
No	24,379 (94.1)	792.7 (761.0, 825.2)	10.0 (9.2, 10.7)	3.6 (2.0, 7.3)
Yes	1515 (5.9)	790.1 (755.8, 824.9) *	9.9 (9.2, 10.6) *	3.8 (2.1, 8.4) *
Body mass index < 30				
No	21,403 (82.7)	785.4 (753.4, 818.1) *	9.9 (9.2, 110.6) *	4.4 (2.3, 8.6) *
Yes	4491 (17.3)	793.9 (762.2, 826.5)	10.0 (9.2, 10.6)	3.5 (1.9, 7.1)
Regular physical activity				
No	6323 (24.4)	793.9 (761.7, 826.4)	9.9 (9.2, 10.7) *	3.7 (2.0, 7.4)
Yes	19,571 (75.6)	792.1 (760.4, 824.7)	10.0 (9.2, 10.7)	3.6 (1.9, 7.3)
Healthy diet				
No	13,303 (51.4)	793.5 (760.9, 826.9)	10.0 (9.2, 10.7)	3.5 (1.8, 7.0)
Yes	12,591 (48.6)	791.8 (760.4, 823.5)	10.0 (9.2, 10.7)	3.8 (2.1, 7.8)
Healthy lifestyle score				
0 (least healthy)	73 (0.3)	770.3 (736.5, 813.3) *	9.7 (9.1, 10.6) *	6.1 (3.6, 9.0) *
1	1178 (4.5)	789.9 (756.6, 824.5)	9.9 (9.2, 10.6)	4.3 (2.3, 8.6)
2	5357 (20.7)	791.4 (758.4, 824.8)	9.9 (9.2, 10.7)	3.7 (2.0, 7.5)
3	11,092 (42.8)	793.9 (761.5, 826.4)	10.0 (9.2, 10.7)	3.5 (1.9, 7.1)
4 (most healthy)	8194 (31.6)	792.2 (761.7, 824.1)	10.0 (9.3, 10.7)	3.7 (2.0, 7.5)

NOTE. * *p*-value from likelihood ratio test is below 0.05. grey matter, hippocampal and white matter hyper intensity volumes are in cubic centimeter (cm^3^). A-level, Advanced level; CSE, Certificate of Secondary Education; NVQ, National Vocational Qualification.

**Table 2:** Differences in grey matter, hippocampal and white matter hyperintensity volumes by genetic AD risk and adherence to healthy lifestyle recommendations in age-combined and age-stratified analyses.

Risk Group		Grey Matter Volume (in cm^3^)		Hippocampal Volume (in cm^3^)		White Matter Hyperintensity (in Log-Transformed cm^3^)	
		(n = 25,894)		(n = 25,883)		(n = 25,025)	
	N *	Beta (95% CI)	*p*	Beta (95% CI)	*p*	Beta (95% CI)	*p*
All							
Favorable lifestyle and low genetic risk	12,829	Reference	Reference	Reference	Reference	Reference	Reference
Unfavorable lifestyle and low genetic risk	4319	−4.92 (−6.14, −3.70)	1.6 × 10^−14^	−0.07 (−0.10, −0.03)	1.2 × 10^−4^	0.11 (0.08, 0.14)	2.4 × 10^−13^
Favorable lifestyle and high genetic risk	6457	0.40 (−0.65, 1.44)	0.46	−0.05 (−0.07, −0.02)	0.002	0.01 (−0.01, 0.04)	0.42
Unfavorable lifestyle and high genetic risk	2289	−4.44 (−6.01, −2.88)	1.7 × 10^−7^	−0.08 (−0.12, −0.03)	4.8 × 10^−4^	0.11 (0.08, 0.15)	7.8 × 10^−9^
p_GRS-interaction_ †		0.88		0.09		0.61	
<60 years							
Favorable lifestyle and low genetic risk	8458	Reference	Reference	Reference	Reference	Reference	Reference
Unfavorable lifestyle and low genetic risk	3059	−4.60 (−6.06, −3.13)	1.7 × 10^−9^	−0.08 (−0.12, −0.04)	1.3 × 10^−4^	0.11 (0.08, 0.15)	7.6 × 10^−10^
Favorable lifestyle and high genetic risk	4392	1.05 (−0.22, 2.33)	0.11	−0.03 (−0.06, −0.01)	0.15	0.01 (−0.02, 0.04)	0.39
Unfavorable lifestyle and high genetic risk	1683	−3.61 (−5.45, −1.77)	3.9 × 10^−4^	−0.05 (−0.10, −0.00)	0.04	0.11 (0.07, 0.15)	1.6 × 10^−6^
p_GRS-interaction_ †		0.96		0.13		0.51	
≥60 years							
Favorable lifestyle and low genetic risk	4371	Reference	Reference	Reference	Reference	Reference	Reference
Unfavorable lifestyle and low genetic risk	1260	−5.49 (−7.72, −3.28)	3.1 × 10^−6^	−0.04 (−0.10, −0.02)	0.24	0.11 (0.06, 0.17)	7.6 × 10^−5^
Favorable lifestyle and high genetic risk	2065	−1.07 (−2.90, 0.76)	0.25	−0.10 (−0.15, −0.05)	1.1 × 10^−4^	0.01 (−0.04, 0.05)	0.76
Unfavorable lifestyle and high genetic risk	606	−6.84 (−9.83, −3.86)	1.8 × 10^−5^	−0.16 (−0.25, −0.08)	1.6 × 10^−4^	0.12 (0.04, 0.20)	0.002
p_GRS-interaction_ †		0.89		0.67		0.85	

NOTE. *p* is the *p*-value for analyses using the healthiest population group (favorable lifestyle and low genetic risk) as a reference in the linear regression. cm^3^ is cubic centimeter. * N distributions per risk groups are slightly lower for analyses involving hippocampal and white matter hyperintensity volumes (Full information including estimates for the association with total brain and white matter volumes found in Table S6). † *p*-value for the interaction term between genetic risk score and healthy lifestyle in the linear regression, with the model adjusted for age, sex, duration until imaging, genotyping array, 40 principal components, education, Townsend deprivation index, employment, alcohol consumption and long-standing illness.

## Data Availability

Data described in the manuscript, code book, and analytic code will be made available upon request and getting approval from UK Biobank.

## Supplementary Materials

The following supporting information can be downloaded at: https://www.mdpi.com/article/10.3390/nu14193907/s1, Text S1: Validation of healthy lifestyle score and AHA (American Heart Association) defined healthy diet; Text S2: Genetic risk score construction; Table S1: Descriptions of lifestyle factors, socioeconomic and demographic variables included in the analyses; Table S2: Validation of the touchscreen-based dietary information using the baseline 24 h recall data; Table S3: List of Alzheimer’s disease (AD) risk increasing variants that were used to construct the genetic risk scores; Table S4: Characteristics of the participants. Table S5: The association between genetic AD risk and neuroimaging outcomes, in basic, socioeconomic and lifestyle factors adjusted models; Table S6: The association of healthy lifestyle and the components with neuroimaging outcomes, in basic, socioeconomic and lifestyle factors adjusted models; Table S7: Differences in total brain, grey matter, white matter, hippocampal and white matter hyperintensity volumes by genetic AD risk and adherence to healthy lifestyle recommendations in age-combined and age-stratified analyses; Figure S1: UK Biobank participants flow for the analyses; Figure S2: The association of healthy lifestyle and its components with mortality; Figure S3: Healthy lifestyle and its components association with brain volumes among all, <60 years, and ≥60 years individuals. Panel A for Total brain, grey matter and white matter volumes, Panel B for Hippocampal volume and white matter hyperintensity volume [6,11,19,22,41,42,43,44,45,46,47,48,49].
