# Supplementary material for: Healthy Lifestyle, Genetic Risk and Brain Health: A Gene-Environment Interaction Study in the UK Biobank
Source: Nutrients. 2022 Sep 21;14(19):3907. doi: 10.3390/nu14193907 (PMC9570683; doi:10.3390/nu14193907)
Supplement: Supplementary file 1 [file nutrients-14-03907-s001.zip › nutrients-1904172-supplementary.pdf]

Supplementary materials

Table of contents

Text S1. Validation of healthy lifestyle score and AHA (American Heart Association) defined healthy diet

Text S2. Genetic risk score construction

Table S1: Descriptions of lifestyle factors, socioeconomic and demographic variables included in the analyses

Table S2. Validation of the touchscreen-based dietary information using the baseline 24-hour recall data.

Table S3. List of Alzheimer's disease (AD) risk increasing variants that were used to construct the genetic risk scores

Table S4. Characteristics of the participants

Table S5. The association between genetic AD risk and neuroimaging outcomes, in basic, socioeconomic and lifestyle factors adjusted models.

Table S6. The association of healthy lifestyle and the components with neuroimaging outcomes, in basic, socioeconomic and lifestyle factors adjusted models.

Table S7. Differences in total brain, grey matter, white matter, hippocampal and white matter hyperintensity volumes by genetic AD risk and adherence to healthy lifestyle recommendations in age-combined and age-stratified analyses.

Figure S1. UK Biobank participants flow for the analyses.

Figure S2. The association of healthy lifestyle and its components with mortality.

Figure S3. Healthy lifestyle and its components association with brain volumes among all, <60 years, and ≥60 years individuals. **Panel A** for Total brain, grey matter and white matter volumes, **Panel B** for Hippocampal volume and white matter hyperintensity volume.

Text S1. Validation of healthy lifestyle score and AHA [American Heart Association] defined healthy diet

Given that some of the lifestyle information was based on self-reported information, *a priori* analyses using mortality as the outcome were carried out to validate the healthy lifestyle score, and its components, with the mortality data obtained from death registry recorded until March 2021. Around 35,000 deaths recorded since the recruitment, and up to 20,642 deaths in unrelated white British ancestry population with complete data on dietary, socioeconomic and lifestyle covariates information were used for the validation analysis. Logistic regression was used to investigate the association of healthy lifestyle score and the components with mortality risk. We used similar list of covariates, and adjustment strategies as the main analysis. We confirmed the expected increases in mortality risk with current smoking (odds ratio [OR] 2.23, 95% CI 2.14, 2.32), and obesity (1.20, 95% CI 1.16, 1.24), and lower risk of mortality with regular exercise (0.84, 95% CI 0.82, 0.87). An overall 'healthy diet' was associated with lower mortality risk (0.92, 95% CI 0.88, 0.95), with a higher consumption of vegetables and fruit and a lower intake of meat (≤2 times processed meat & ≤5 times red meat intake per week) contributing to lower risk of mortality (Figure S2). The AHA definition of a healthy diet also included a measure of fish intake, which in this population was not associated with mortality (Figure S2), which might be due to the traditional British dish 'fish and chips' is made of white fish, commonly deep fried, and prepared in a way that it is high in saturated fat, which in turn has been associated with a higher risk of dementia (1) and reduced brain volumes (2). The composite scores of the four lifestyles, referred in the study as healthy lifestyle, associated with lower risk of mortality (0.75, 95% CI 0.74, 0.76 per unit higher healthy lifestyle score).

For items in the AHA defined healthy diet, we confirmed the associations (using linear regression) between food frequency information from the touchscreen questionnaire against data on approximate intakes (grams/day) calculated based on baseline 24-hour recall which was available for a subsample of UK Biobank participants. The dietary components from food frequency were strongly associated with the respective information from 24-hour recall (Table S2).

Text S2. Genetic variant selection and risk score construction

We selected genetic variants from the most recent GWAS that did not include the UK Biobank (4) and which based their case-control definition on clinical or pathological assessments (4). This study identified 22 independent single nucleotide polymorphisms (SNPs) associated with an increased AD-risk, and the risk score of these variants associated with higher risk of dementia (OR 2.22, 95% CI 1.79, 2.74 per 10 allele higher in risk score) and lower memory score (beta -0.07, 95%CI -0.09, -0.05) (5). We extracted these variants from UK Biobank 3rd release genome-wide data, and each variant was coded as 0,1 or 2 as per the AD-risk allele. We generated a weighted risk score by the summing the products of number of risk alleles and weight of each allele, with weight is the variant-AD association estimates taken from the discovery meta-analysis of genome-wide association studies (4). We further scaled the risk score by the ratio of the available number of available variants to the sum of weights to express the association estimates per AD risk increasing alleles (*equation 1*).

Equation 1

$$\text{Weighted genetic risk score (GRS)} = \frac{(\beta_1 \text{ x } SNP_1 + \beta_2 \text{ x } SNP_2 + \ldots \beta_{22} \text{ x } SNP_{22}) \text{ x } N_{SNPs}}{\text{Sum of } \beta \text{ coefficients}}$$

Where: β_1 to β_{22} is a coefficient from SNP-AD association estimates from discovery GWAS, SNP_1 to SNP_{22} are the number of AD risk increasing alleles for each SNP and N_{SNPs} is the available number of SNPs per individuals.

Table S1. Descriptions of lifestyle factors, socioeconomic and demographic variables included in the analyses*

Covariate	Categorisation or description [as used]	UK Biobank field ID	Remarks
**Sex**	Male<br>Female	31 [Self-reported sex at recruitment]	
**Age**	40-49 years<br>50-59 years<br>60-73 years	21022 [Age at recruitment]	
**Assessment centre**	Stockport [Cheadle] (of 59.7% of data), Newcastle (25.5%), Reading (14.7), Bristol (0.1%)	54 [UK Biobank Assessment Centres]	Imaging data were collected only from four of the 22 UK Biobank assessment centres.
**Duration until imaging**	Duration in years between the baseline and imaging visits date	53_0 [baseline visit date indicator]<br>53_2 [imaging visit date indicator]	
**Education**	None<br>Intermediate (NVQ/CSE/A-levels)<br>High (degree/professional)	6138 and 10722 (pilot) [“Which of the following qualifications do you have?”]	From touchscreen questionnaires collected at baseline visit
**Employment status**	Unemployed<br>Retired<br>Lowest working hour (first quartile)<br>Second quartile<br>Third quartile<br>Highest working hour (fourth quartile)	6142 and 20119 (pilot) [Which of the following describes your current (“working”) situation?<br>767 [In a typical WEEK, how many hours do you spend at work? (Do not include hours travelling to and from work"	From touchscreen questionnaire collected at the baseline visit
**Townsend deprivation index (TDI)**	Low (higher socioeconomic status) Vs High deprivation index (lower socioeconomic status) using median	189 [Townsend deprivation index calculated immediately prior to participating to participant joining the UK Biobank, based on the preceding national census output areas]	Calculated based on car ownership, household overcrowding, owner occupation, and unemployment for the information available at residential area level
**Alcohol consumption**	Non-drinker<br>Special occasions<br>1-3 times/month<br>1 or 2 times/week<br>3 0r 4 times/week<br>Daily or almost daily	1558 ["About how often do you drink alcohol?"]	
**Smoking**	Non-smokers<br>Ex-smokers<br>Current smokers	20116 [Current/past smoking status]	Non-smokers and ex-smoker codes as 1 and current smokers as 0 for the healthy lifestyle score
**Regular physical activity**	Yes<br>No	884 [In a typical WEEK, on how many days did you do 10 minutes or more of moderate physical activities like carrying light loads, cycling at a normal pace? (Do not include walking)]<br>894 [How many minutes did you usually spend doing moderate activities on a typical DAY?]<br><br>904 [In a typical WEEK, how many days did you do 10 minutes or more of vigorous physical activity? (These are activities that make you sweat or breathe hard such as fast cycling, aerobics, heavy lifting)]<br>914 ["How many minutes did you usually spend doing vigorous activities on a typical DAY?"]	Classification was based on AHA recommended physical activity of (11), and individuals who met either of the below criteria were coded “Yes” otherwise “No” for the regular physical activity.<br>- Moderate physical activity at least for 150 minute per week<br>OR<br>- Vigorous physical activity for at least 75 minutes per week<br>OR<br>- Moderate physical activity for at least five days<br>OR<br>- Vigorous physical activity for at more than one day<br>OR<br>- Combination of the above<br><br>Those with recommend regular activity coded 1 otherwise 0 for the healthy lifestyle score.
**Body mass index**	<18.5 kg/m^2<br>[18.5, 25) kg/m^2<br>[25,30) kg/ m^2<br>>=30kg/m^2	21002 [Measured weight]<br>50 [Standing height measure using a Seca 202 device]	Body mass index [BMI] derived as weight (in Kg) over height (in meter) square. BMI of 30 kg/m^2 and above coded as 0 and those below that as 1 for the healthy lifestyle score.
**Healthy diet**	At least two healthy food items Vs Fewer than two healthy food items	1289 [On average how many heaped tablespoons of COOKED vegetables would you eat per DAY?]<br>1299 [On average how many heaped tablespoons of SALAD or RAW vegetables would you eat per DAY]<br>1309 [About how many pieces of FRESH fruit would you eat per DAY? (Count one apple, one banana, 10 grapes etc as one piece; put '0' if you do not eat any)]<br><br>1329 [How often do you eat oily fish? (e.g., sardines, salmon, mackerel, herring)]<br>1339 [How often do you eat other types of fish? (e.g., cod, tinned tuna, haddock)]<br><br>1349 [How often do you eat processed meats (such as bacon, ham, sausages, meat pies, kebabs, burgers, chicken nuggets)?]<br><br>1369 [How often do you eat beef?]<br>1379 [How often do you eat lamb/mutton?]<br>1389 [How often do you eat pork? (Do not count processed meats such as bacon or ham)]	Healthy diet constructed based on the frequency/amount of food items intake collected using touchscreen questionnaire. Healthy diet constructed based on a method described in prior study (12), adopted from AHA (13)<br>Each of the below criteria scored 1, and summed to generate healthy diet with range 0 to 4:<br>- Total fruit and vegetable intake were ≥4.5 pieces OR serving (1 serving = ~3 tablespoons vegetables) per day<br>- Total fish intake ≥2 times per week<br>- Processed meat intake <2 times a week<br>- Red meet intake <5 times a week<br><br>The higher the score the healthier the diet, and those with score of two and above coded as 1 otherwise 0 for the healthy lifestyle score.
**Longstanding illness/infirmity /disability**	Yes<br>No	2188 [Do you have any long-standing illness, disability or infirmity?]	

* Information used were those collected at recruitment/baseline visits. NVQ, National Vocational Qualification; CSE, Certificate of Secondary Education; A-levels, Advanced Levels. AHA, American Heart Association. Information can be found in the UK Biobank data showcase (https://www.ukbiobank.ac.uk/). Light green shaded regions contributed for deriving healthy lifestyle score, with being non obese (<30kg/m^2), not currently smoking, regularly exercise, and had at least two healthy food items had each score of one and summed together to generate healthy lifestyle score with ranges of zero to four.

Table S2. Validation of the touchscreen-based dietary information using the baseline 24-hour recall data.

Food intake (grams per day) from **24-hour recall**	**Food frequency** from touchscreen questionnaire	n	beta	LCI	UCI	P
Fruit and vegetables	Vegetable & fruit (≥4.5 servings)	4,983	249.21	231.73	266.69	0.0E+00
Fish	Total fish (≥2 times per week)	4,983	21.92	18.89	24.94	0.0E+00
Red meat	Low meat (≤2 times processed & ≤5 times red meat per week)	4,981	-25.44	-30.64	-20.24	1.4E-21

Dietary consumption of selected food items based on 24-hour recall, presented as grams per day. Estimates in grams per day from linear regression, after adjusting for age, sex, assessment centre, duration until imaging (in years), education, Townsend deprivation index, employment, obesity, smoking, alcohol consumption, physical activity and long-standing illness. The analysis was restricted to the imaging analysis sample with complete touchscreen-based food frequency and baseline 24-hour recall dietary information (n= up to 4,983).

Table S3. List of Alzheimer's disease (AD) risk increasing variants that were used to construct the genetic risk scores

SNP	Chr.	Pos.	closest gene	EA	OA	EAF	n	logOR	SE_logOR	p	INFO SCORE$_{UKB}$	EAF$_{UKB}$
rs6656401	1	207692049	CR1	A	G	0.197	74,046	0.1667	0.0165	5.7E-24	0.9991	0.172
rs35349669	2	234068476	INPP5D	T	C	0.488	74,046	0.0755	0.0136	3.2E-08	0.9968	0.483
rs6733839	2	127892810	BIN1	T	C	0.409	74,046	0.1965	0.0141	6.9E-44	0.9635	0.391
rs190982	5	88223420	MEF2C	A	G	0.592	74,046	0.0759	0.0137	3.2E-08	0.9674	0.609
rs10948363	6	47487762	CD2AP	G	A	0.266	74,046	0.0954	0.0145	5.2E-11	0.9992	0.271
rs9271192	6	32579070	HLA-DRB5-HLA-DRB1	C	A	0.276	74,046	0.1069	0.016	2.7E-11	0.9998	0.274
rs11771145	7	143110762	EPHA1	G	A	0.662	74,046	0.102	0.0137	1.1E-13	1	0.649
rs1476679	7	100004446	ZCWPW1	T	C	0.713	74,046	0.0891	0.0144	5.6E-10	0.9974	0.698
rs2718058	7	37841534	NME8	A	G	0.627	74,046	0.0774	0.0132	4.8E-09	0.9937	0.637
rs28834970	8	27195121	PTK2B	C	T	0.366	74,046	0.0996	0.0133	7.4E-14	0.9962	0.365
rs9331896	8	27467686	CLU	T	C	0.621	74,046	0.146	0.0141	2.8E-25	0.9895	0.588
rs10792832	11	85867875	PICALM	G	A	0.642	74,046	0.14	0.0133	9.3E-26	0.9993	0.634
rs10838725	11	47557871	CELF1	C	T	0.316	74,046	0.0789	0.0138	1.1E-08	0.9994	0.300
rs11218343	11	121435587	SORL1	T	C	0.961	74,046	0.262	0.0338	9.7E-15	0.9923	0.960
rs983392	11	59923508	MS4A6A	A	G	0.597	74,046	0.1081	0.0134	6.1E-16	0.9944	0.599
rs10498633	14	92926952	SLC24A4, RIN3	G	T	0.783	74,046	0.0946	0.0162	5.5E-09	1	0.773
rs17125944	14	53400629	FERMT2	C	T	0.092	74,046	0.1323	0.0229	7.9E-09	0.9916	0.092
rs8093731	18	29088958	DSG2	C	T	0.983	74,046	0.6136	0.1123	4.6E-08	1	0.980
rs3865444	19	51727962	CD33	C	A	0.693	74,046	0.0954	0.0175	5.1E-08	1	0.686
rs4147929	19	1063443	ABCA7	A	G	0.19	74,046	0.143	0.0178	1.1E-15	0.9975	0.174
rs429358	19	45411941	APOE	C	T	0.17	74,046	1.3503	0.0272	1.0E-200	1	0.154
rs7274581	20	55018260	CASS4	T	C	0.917	74,046	0.1323	0.0237	2.5E-08	0.9860	0.914

Genetic variants (Single nucleotide polymorphism [SNP]) are from the latest meta-analysis of genome-wide association studies for Alzheimer's disease that did not include participants of the UK Biobank (UKB), with effect allele frequency (EAF) and estimates in the light blue shaded region from the discovery study (4). Exponentiating logOR is equivalent with odds ratio (OR). Imputation quality (INFO SCORE) and the respective effect allele frequency (EAF$_{UKB}$) from the UKB are shown in the grey shaded region.

Table S4. Characteristics of the participants

	Baseline sample[1] n (%)	Brain volume analytic sample[2], n (%)
All	308,846	25,894
Sex		
Men	143,001 (46.3)	12,286 (47.4)
Women	165,845 (53.7)	13,608 (52.6)
Age		
39 – 49 years	68,719 (22.3)	6,839 (26.4)
50 – 59 years	102,563 (33.2)	10,753 (41.5)
60 – 73 years	137,564 (44.5)	8,302 (32.1)
Education		
None	48,552 (15.7)	1,535 (5.9)
NVQ/CSE/A-levels	110,857 (35.9)	8,071 (31.2)
Degree/professional	149,437 (48.4)	16,288 (62.9)
Employment		
None	23,476 (7.6)	1,493 (5.8)
Retired	107,855 (34.9)	6,580 (25.4)
Lowest working hour (1st quartile)	43,718 (14.2)	4,085 (15.8)
2nd quartile	30,079 (9.7)	2,999 (11.6)
3rd quartile	55,929 (18.1)	5,732 (22.1)
Highest working hour (4th quartile)	47,789 (15.5)	5,005 (19.3)
Townsend deprivation index		
Highly deprived	140,946 (45.6)	10,232 (39.5)
Less deprived	167,900 (54.4)	15,662 (60.5)
Alcohol		
Non-drinker	19,030 (6.2)	1,040 (4.0)
Special occasion only	31,059 (10.1)	1,929 (7.4)
1-3 times/month	34,058 (11.0)	2,737 (10.6)
1-2 times/week	81,081 (26.3)	6,682 (25.8)
3-4 times/week	76,080 (24.6)	7,527 (29.1)
Daily or almost daily	67,538 (21.9)	5,979 (23.1)
Long standing illness		
No	210,879 (68.3)	19,920 (76.9)
Yes	97,967 (31.7)	5,974 (23.1)
Healthy lifestyle factors		
No current smoking	278,992 (90.3)	24,379 (94.2)
Body mass index < 30	236,248 (76.5)	21,403 (82.7)
Regular physical activity	227,030 (73.5)	19,571 (75.6)
Healthy diet	151,317 (48.9)	12,591 (48.6)
Healthy lifestyle score		
0 (least healthy)	1,819 (0.6)	73 (0.3)
1	21,276 (6.9)	1,178 (4.5)
2	72,172 (23.4)	5,357 (20.7)
3	126,370 (40.9)	11,092 (42.8)
4 (most healthy)	87,209 (28.2)	8,194 (31.6)

Characteristics of the participants for the sample size [1]in the baseline assessment (unrelated white British participants complete genetic, lifestyle and socioeconomic information, and no history of dementia) Vs.[2]in the brain imaging analysis (restricted to those with complete neuroimaging information, with further exclusion of those with outlier neuroimaging data). The average time interval between baseline and imaging visits was 8.8 years (1.7 SD).

Table S5. The association between genetic AD risk and neuroimaging outcomes, in basic, socioeconomic and lifestyle factors adjusted models.

		**Basic**				**Basic + Socioeconomic**				**Basic + Socioeconomic + lifestyle factors + longstanding illness**				
Outcome	n	Beta	LCI	UCI	P	Beta	LCI	UCI	P	Beta	LCI	UCI	P	$p_{age\text{-}interaction}$*
Total brain volume (cm^3)	25,894	0.25	-1.61	2.12	0.79	0.24	-1.62	2.10	0.80	0.36	-1.50	2.21	0.71	**0.003**
Grey matter volume (cm^3)	25,894	0.44	-0.68	1.56	0.44	0.42	-0.70	1.54	0.47	0.49	-0.63	1.60	0.39	**0.007**
White matter volume (cm^3)	25,894	-0.19	-1.39	1.02	0.76	-0.17	-1.38	1.03	0.78	-0.13	-1.33	1.07	0.83	0.02
Hippocampal volume (cm^3)	25,883	**-0.06**	**-0.09**	**-0.03**	**4.6×10^{-5}**	**-0.06**	**-0.09**	**-0.03**	**4.9×10^{-5}**	**-0.06**	**-0.09**	**-0.03**	**6.3×10^{-5}**	**6.1×10^{-5}**
White matter hyperintensity volume (log-transformed cm^3)†	25,025	0.03	-0.00	0.05	0.06	0.03	0.00	0.06	0.06	0.03	0.00	0.05	0.06	0.76

*p-value for the interaction between age and genetic AD risk, those in bold are below the multiple test corrected threshold (p<0.01).

†White matter hyperintensity volume were log transformed to approximate the normal distribution.

Table S6. The association of healthy lifestyle and the components with neuroimaging outcomes, in basic, socioeconomic and lifestyle factors adjusted models.

			Basic				Basic + Socioeconomic				Basic + Socioeconomic + lifestyle factors + longstanding illness					
Outcome	Exposure	n	Beta	LCI	UCI	P	Beta	LCI	UCI	P	Beta	LCI	UCI	P	$p_{age\text{-}interaction}$*	$p_{GRS\text{-}interaction}$†
Total brain volume (cm^3)	**Healthy lifestyle**	25,894	**2.32**	**1.48**	**3.15**	**5.5E-08**	**2.51**	**1.67**	**3.35**	**4.8E-09**	**2.43**	**1.59**	**3.28**	**1.5E-08**	0.85	0.14
	Current smoking	25,894	**-9.62**	**-12.65**	**-6.60**	**4.6E-10**	**-9.84**	**-12.87**	**-6.80**	**2.1E-10**	**-9.37**	**-12.39**	**-6.34**	**1.3E-09**	0.51	0.78
	Obesity	25,894	**-8.29**	**-10.16**	**-6.42**	**3.9E-18**	**-8.61**	**-10.49**	**-6.73**	**2.7E-19**	**-8.91**	**-10.80**	**-7.01**	**4.1E-20**	0.02	0.69
	Regular Physical activity	25,894	0.89	-0.76	2.55	0.29	1.26	-0.39	2.92	0.14	0.23	-1.44	1.90	0.79	0.51	0.04
	Healthy diet	25,894	-0.94	-2.38	0.50	0.20	-0.89	-2.33	0.55	0.23	-1.26	-2.70	0.18	0.09	0.05	0.28
Grey matter volume (cm^3)	**Healthy lifestyle**	25,894	**2.41**	**1.90**	**2.92**	**1.1E-20**	**2.48**	**1.97**	**2.99**	**1.2E-21**	**2.45**	**1.94**	**2.96**	**4.5E-21**	0.45	0.58
	Current smoking	25,894	**-8.40**	**-10.23**	**-6.57**	**2.7E-19**	**-8.42**	**-10.26**	**-6.59**	**2.6E-19**	**-8.22**	**-10.04**	**-6.40**	**9.4E-19**	0.31	0.61
	Obesity	25,894	**-8.94**	**-10.07**	**-7.81**	**0.0E+00**	**-9.03**	**-10.16**	**-7.90**	**0.0E+00**	**-9.18**	**-10.32**	**-8.04**	**0.0E+00**	0.80	0.43
	Regular Physical activity	25,894	0.89	-0.11	1.89	0.08	1.03	0.03	2.04	0.04	-0.01	-1.02	0.99	0.98	0.20	0.36
	Healthy diet	25,894	-0.69	-1.57	0.18	0.12	-0.66	-1.53	0.21	0.14	-0.97	-1.83	-0.10	0.03	0.01	0.89
White matter volume (cm^3)	**Healthy lifestyle**	25,894	-0.09	-0.63	0.45	0.74	0.03	-0.52	0.57	0.92	-0.02	-0.56	0.53	0.94	0.28	0.08
	Current smoking	25,894	-1.22	-3.18	0.73	0.22	-1.41	-3.37	0.55	0.16	-1.14	-3.11	0.82	0.25	0.95	0.36
	Obesity	25,894	0.65	-0.56	1.86	0.29	0.42	-0.80	1.63	0.50	0.28	-0.96	1.51	0.66	**3.90E-04**	0.2
	Regular Physical activity	25,894	0.00	-1.06	1.07	0.99	0.23	-0.84	1.30	0.68	0.24	-0.84	1.33	0.66	0.82	0.02
	Healthy diet	25,894	-0.25	-1.18	0.68	0.60	-0.23	-1.16	0.70	0.63	-0.30	-1.23	0.64	0.54	0.48	0.13
Hippocampal volume (cm^3)	**Healthy lifestyle**	25,883	**0.03**	**0.02**	**0.04**	**2.6E-05**	**0.03**	**0.02**	**0.05**	**6.6E-06**	**0.03**	**0.02**	**0.05**	**1.4E-05**	0.90	0.82
	Current smoking	25,883	**-0.10**	**-0.15**	**-0.05**	**1.3E-04**	**-0.10**	**-0.15**	**-0.05**	**1.0E-04**	**-0.10**	**-0.15**	**-0.05**	**1.2E-04**	0.29	0.28
	Obesity	25,883	**-0.08**	**-0.12**	**-0.05**	**1.0E-07**	**-0.09**	**-0.12**	**-0.06**	**2.1E-08**	**-0.08**	**-0.12**	**-0.05**	**2.1E-07**	0.75	0.36
	Regular Physical activity	25,883	0.04	0.01	0.06	0.009	0.04	0.01	0.07	0.003	0.03	0.00	0.06	0.03	0.64	0.98
	Healthy diet	25,883	-0.01	-0.03	0.01	0.37	-0.01	-0.04	0.01	0.34	-0.02	-0.04	0.01	0.17	0.59	0.73
White matter hyperintensity volume (Log-transformed cm^3)	**Healthy lifestyle**	25,025	**-0.07**	**-0.08**	**-0.06**	**1.7E-26**	**-0.07**	**-0.08**	**-0.05**	**3.5E-25**	**-0.06**	**-0.08**	**-0.05**	**2.6E-22**	0.63	0.58
	Current smoking	25,025	**0.24**	**0.19**	**0.28**	**2.7E-24**	**0.23**	**0.18**	**0.27**	**1.1E-22**	**0.23**	**0.19**	**0.28**	**1.4E-23**	0.06	0.31
	Obesity	25,025	**0.22**	**0.19**	**0.25**	**0.0E+00**	**0.22**	**0.19**	**0.25**	**0.0E+00**	**0.20**	**0.18**	**0.23**	**0.0E+00**	**2.50E-04**	0.35
	Regular Physical activity	25,025	**-0.04**	**-0.06**	**-0.01**	**0.002**	**-0.04**	**-0.06**	**-0.02**	**0.002**	-0.01	-0.04	0.01	0.40	0.42	0.92
	Healthy diet	25,025	0.01	-0.01	0.03	0.45	0.01	-0.01	0.03	0.34	0.02	0.00	0.04	0.08	0.02	0.38

Estimates are from linear regression adjusted for basic (age, sex, assessment centre, duration until imaging (in years)), socioeconomic (education, Townsend deprivation index, and employment.), lifestyle factors (components of healthy lifestyle factor and alcohol consumption), and long-standing illness. GRS-lifestyle factors interaction analyses were further adjusted for 40 principal components and genotyping array

*p-value for the interaction between age and lifestyle factors, generated from the final model.

†p-value the interaction between AD genetic risk score and lifestyle factors, generated from the final model.

5.5E-08 expression is similar as 5.5 x 10^{-8}

Table S7. Differences in total brain, grey matter, white matter, hippocampal and white matter hyperintensity volumes by genetic AD risk and adherence to healthy lifestyle recommendations in age-combined and age-stratified analyses.

	**Total brain volume in cm³ (n=25,894)**			**Grey matter volume in cm³ (n=25,894)**			**White matter volume in cm³ (n=25,894)**			**Hippocampal volume in cm³ (n=25,883)**			**White matter hyperintensity volume in log-transformed cm³ (n=25,025)**		
Risk group	n	Beta (95% CI)	p	n	Beta (95% CI)	p	n	Beta (95% CI)	p	n	Beta (95% CI)	p	n	Beta (95% CI)	p
**All**															
Favorable lifestyle and low genetic risk	12,829	Reference	Reference	12,829	Reference	Reference	12,829	Reference	Reference	12,825	Reference	Reference	12,400	Reference	Reference
Unfavorable lifestyle and low genetic risk	4,319	-5.39 (-7.41, -3.38)	3.10E-07	4,319	-4.92 (-6.14, -3.70)	1.60E-14	4,319	-0.47 (-1.78, 0.83)	0.48	4,318	-0.07 (-0.10, -0.03)	1.20E-04	4,186	0.11 (0.08, 0.14)	2.40E-13
Favorable lifestyle and high genetic risk	6,457	0.36 (-1.36, 2.10)	0.81	6,457	0.40 (-0.65, 1.44)	0.46	6,457	-0.03 (-1.14, 1.10)	0.96	6,451	-0.05 (-0.07, -0.02)	0.002	6,235	0.01 (-0.01, 0.04)	0.42
Unfavorable lifestyle and high genetic risk	2,289	-4.41 (-6.69, -1.82)	0.002	2,289	-4.44 (-6.01, -2.88)	1.70E-07	2,289	0.04 (-1.64, 1.71)	0.97	2,288	-0.08 (-0.12, -0.03)	4.80E-04	2,204	0.11 (0.08, 0.15)	7.80E-09
$p_{GRS\text{-}interaction}$*		0.84			0.88			0.66			0.09			0.61	
**<60 years**															
Favorable lifestyle and low genetic risk	8,458	Reference	Reference	8,458	Reference	Reference	8,458	Reference	Reference	8,457	Reference	Reference	8,174	Reference	Reference
Unfavorable lifestyle and low genetic risk	3,059	-5.59 (-8.03, -3.17)	8.10E-06	3,059	-4.60 (-6.06, -3.13)	1.70E-09	3,059	-0.99 (-2.53, 0.54)	0.20	3,058	-0.08 (-0.12, -0.04)	1.30E-04	2,968	0.11 (0.08, 0.15)	7.60E-10
Favorable lifestyle and high genetic risk	4,392	1.36 (-0.76, 3.49)	0.21	4,392	1.05 (-0.22, 2.33)	0.11	4,392	0.31 (-1.02, 1.64)	0.65	4,388	-0.03 (-0.06, -0.01)	0.15	4,255	0.01 (-0.02, 0.04)	0.39
Unfavorable lifestyle and high genetic risk	1,683	-3.43 (-6.50, -0.38)	0.05	1,683	-3.61 (-5.45, -1.77)	3.90E-04	1,683	0.17 (-1.75, 2.10)	0.86	1,683	-0.05 (-0.10, -0.00)	0.04	1,625	0.12 (0.07, 0.16)	1.60E-06
$p_{GRS\text{-}interaction}$*		0.70			0.96			0.51			0.13			0.62	
**≥60 years**															
Favorable lifestyle and low genetic risk	4,371	Reference	Reference	4,371	Reference	Reference	4,371	Reference	Reference	4,368	Reference	Reference	4,226	Reference	Reference
Unfavorable lifestyle and low genetic risk	1,260	-4.37 (-7.99, -0.74)	0.02	1,260	-5.49 (-7.72, -3.28)	3.10E-06	1,260	1.13 (-1.35, 3.61)	0.34	1,260	-0.04 (-0.10, -0.02)	0.24	1,218	0.11 (0.06, 0.17)	7.60E-05
Favorable lifestyle and high genetic risk	2,065	-1.92 (-4.91, 1.07)	0.21	2,065	-1.07 (-2.90, 0.76)	0.25	2,065	-0.85 (-2.90, 1.20)	0.39	2,064	-0.10 (-0.15, -0.05)	1.10E-04	1,980	0.01 (-0.04, 0.05)	0.76
Unfavorable lifestyle and high genetic risk	606	-7.41 (-12.29, -2.53)	0.003	606	-6.84 (-9.83, -3.86)	1.80E-05	606	-0.57 (-3.91, 2.78)	0.78	605	-0.16 (-0.25, -0.08)	1.60E-04	579	0.12 (0.04, 0.20)	0.002
$p_{GRS\text{-}interaction}$*		0.73			0.89			0.70			0.67			0.99	

p is the p-value for analysis using the healthiest population group (favorable lifestyle and low genetic risk) as a reference in the linear regression. 3.1E-07 expression is similar as 3.1×10^{-7}

*p-value for the interaction term between genetic risk score and healthy lifestyle in the linear regression, with the model adjusted for age, sex, duration until imaging, genotyping array, 40 principal components, education, Townsend deprivation index, employment, and long-standing illness.

p-value for three-way interactions between age, healthy lifestyle, and genetic AD risk was ≥ 0.62, for all

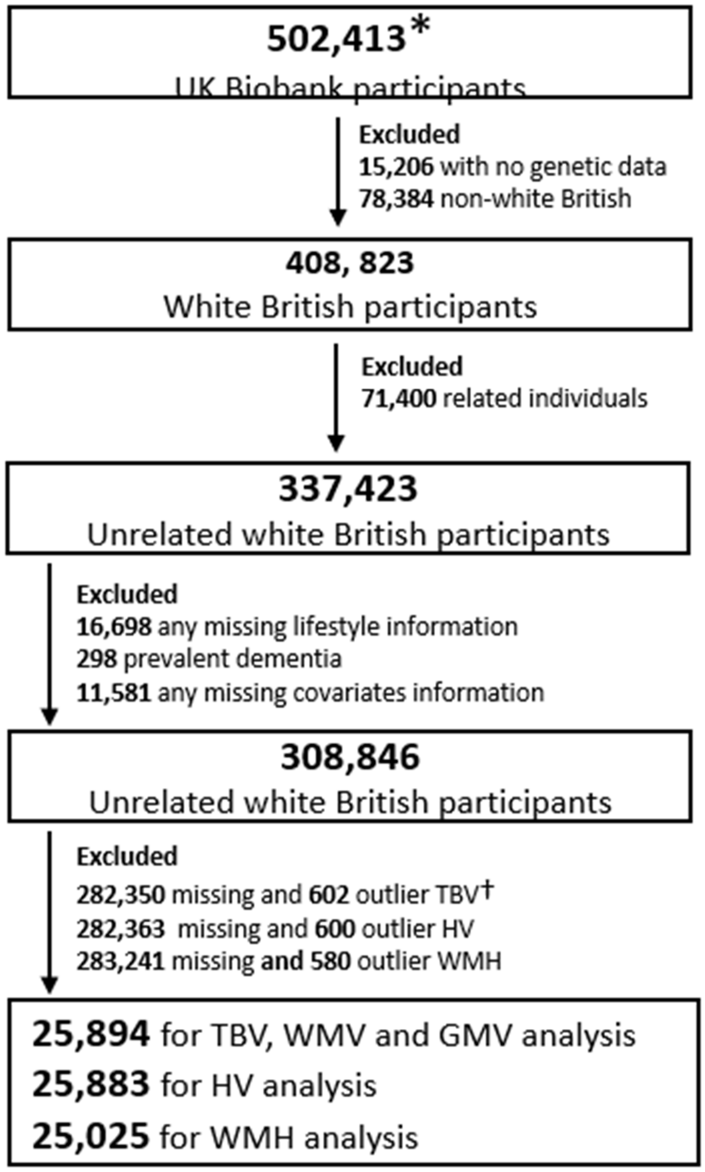

Figure S1. UK Biobank participants flow for the analyses.

* Participants with active consent.

† Exclusion criteria works also for GMV and WMV.

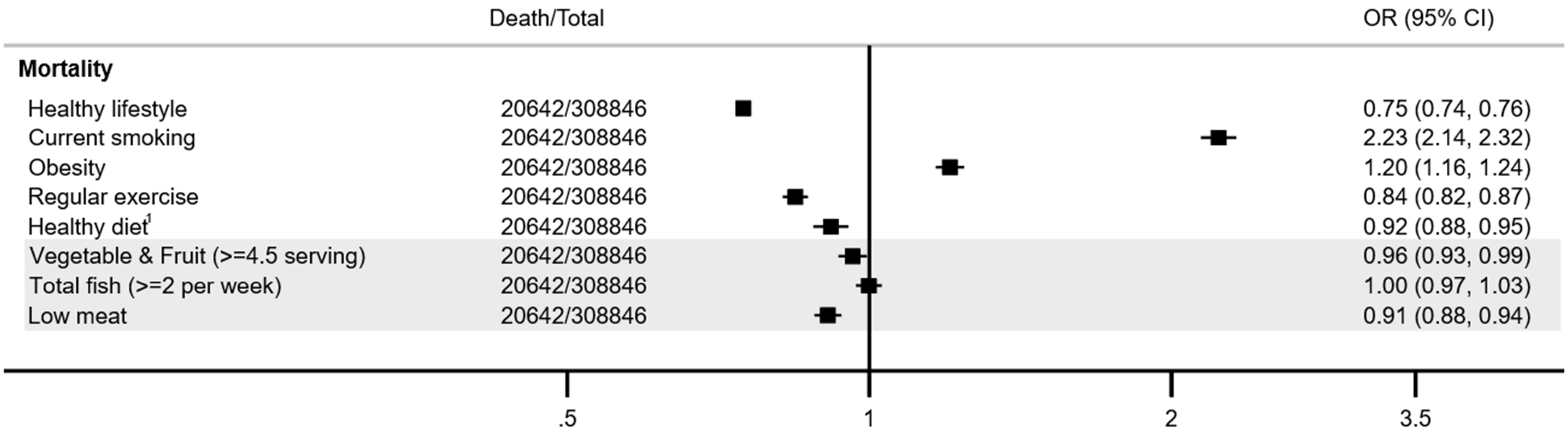

Figure S2. The association of healthy lifestyle and its components with mortality.

Estimates are from a logistic regression in a model adjusted for age, sex, assessment centre, duration until imaging (in years), education, Townsend deprivation index, employment, components of healthy lifestyle (exception for healthy lifestyle analyses), alcohol consumption and long-standing illness.

Panel A

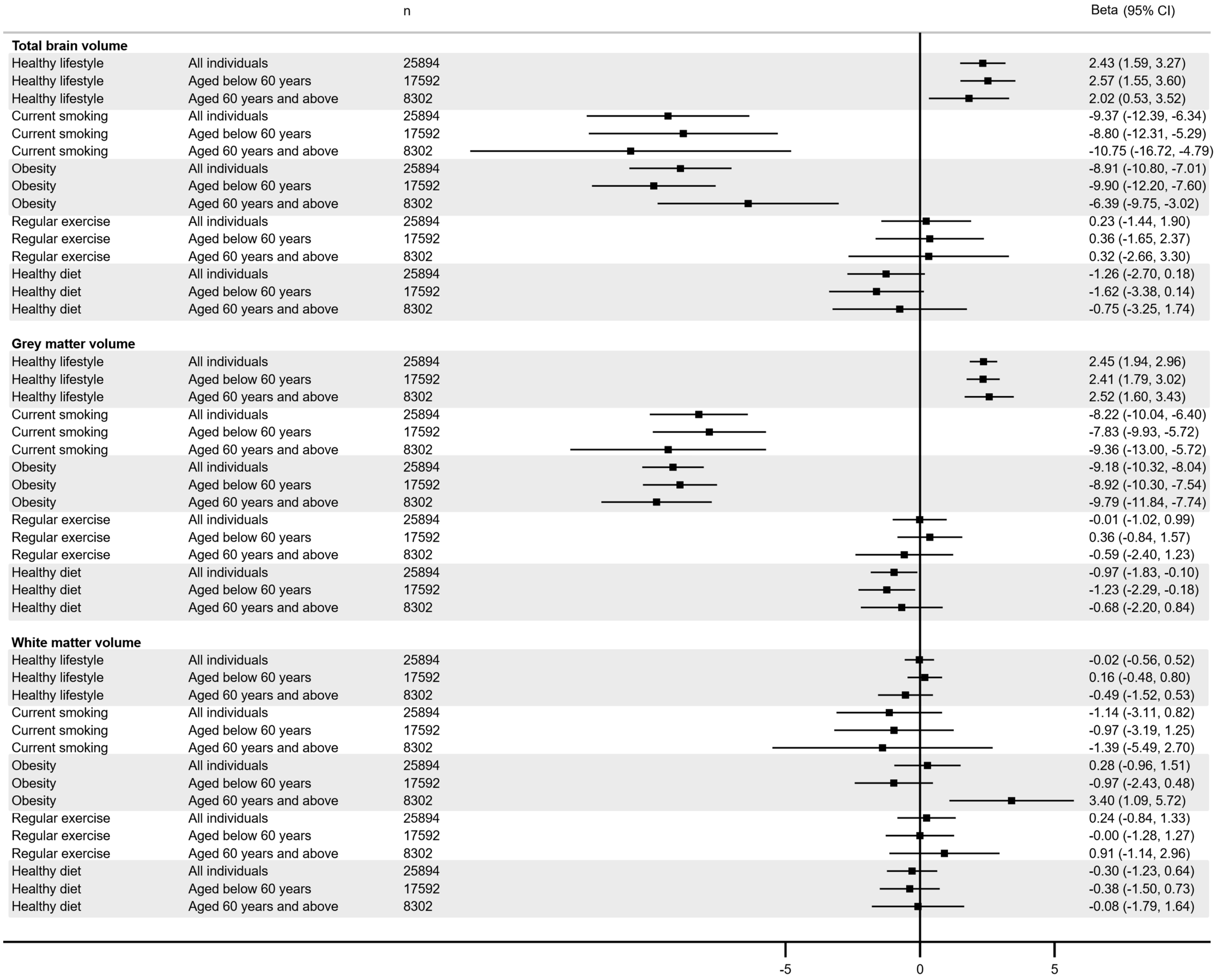

Panel B

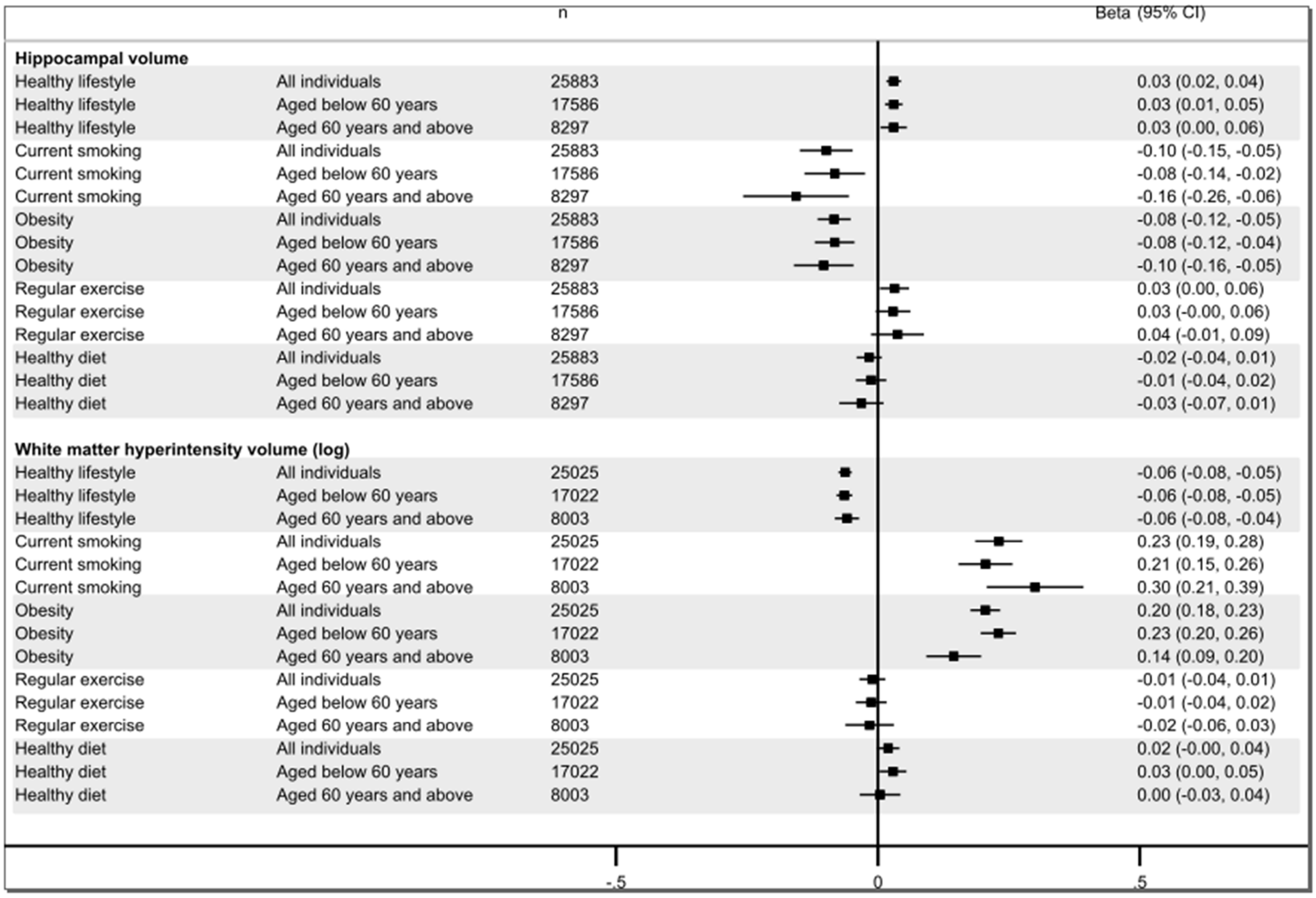

Figure S3. Healthy lifestyle and its components association with brain volumes among all, <60 years, and ≥60 years individuals. **Panel A** for Total brain, grey matter and white matter volumes (in cm^3), **Panel B** for Hippocampal volume (in cm^3) and white matter hyperintensity volume (in log-transformed cm^3).

Estimates from a model adjusted for age, sex, assessment centre, duration until imaging (in years) education, Townsend deprivation, employment, components of healthy lifestyle (exception health lifestyle analyses), alcohol consumption and long-standing illness.

References

1. Barbaresko J, Lellmann AW, Schmidt A, Lehmann A, Amini AM, Egert S, Schlesinger S, Nöthlings U. Dietary Factors and Neurodegenerative Disorders: An Umbrella Review of Meta-Analyses of Prospective Studies. Advances in Nutrition 2020;11(5):1161-73.
2. Jensen DE, Leoni V, Klein-Flügge MC, Ebmeier KP, Suri S. Associations of dietary markers with brain volume and connectivity: A systematic review of MRI studies. Ageing research reviews 2021:101360.
3. Paulson HL, Igo I. Genetics of dementia. Semin Neurol 2011;31(5):449-60. doi: 10.1055/s-0031-1299784.
4. Lambert JC, Ibrahim-Verbaas CA, Harold D, Naj AC, Sims R, Bellenguez C, DeStafano AL, Bis JC, Beecham GW, Grenier-Boley B, et al. Meta-analysis of 74,046 individuals identifies 11 new susceptibility loci for Alzheimer's disease. Nat Genet 2013;45(12):1452-8. doi: 10.1038/ng.2802.
5. Marden JR, Walter S, Tchetgen Tchetgen EJ, Kawachi I, Glymour MM. Validation of a polygenic risk score for dementia in black and white individuals. Brain and behavior 2014;4(5):687-97. doi: 10.1002/brb3.248.
6. Novak G, Einstein SG. Chapter 4 - structural magnetic resonance imaging as a biomarker for the diagnosis, progression, and treatment of alzheimer disease. In: McArthur RA, ed. Translational Neuroimaging. 1st ed. London: Academic Press, 2013:87-129.
7. Vemuri P, Jack CR. Role of structural MRI in Alzheimer's disease. Alzheimer's Research & Therapy 2010;2(4):23. doi: 10.1186/alzrt47.
8. Banerjee D, Muralidharan A, Hakim Mohammed AR, Malik BH. Neuroimaging in Dementia: A Brief Review. Cureus 2020;12(6):e8682-e. doi: 10.7759/cureus.8682.
9. Dicks E, Vermunt L, van der Flier WM, Visser PJ, Barkhof F, Scheltens P, Tijms BM. Modeling grey matter atrophy as a function of time, aging or cognitive decline show different anatomical patterns in Alzheimer's disease. NeuroImage: Clinical 2019;22:101786. doi: https://doi.org/10.1016/j.nicl.2019.101786.
10. Hase Y, Horsburgh K, Ihara M, Kalaria RN. White matter degeneration in vascular and other ageing-related dementias. Journal of neurochemistry 2018;144(5):617-33. doi: 10.1111/jnc.14271.
11. Lloyd-Jones DM, Hong Y, Labarthe D, Mozaffarian D, Appel LJ, Van Horn L, Greenlund K, Daniels S, Nichol G, Tomaselli GF, et al. Defining and setting national goals for cardiovascular health promotion and disease reduction: the American Heart Association's strategic Impact Goal through 2020 and beyond. Circulation 2010;121(4):586-613. doi: 10.1161/circulationaha.109.192703.
12. Rutten-Jacobs LC, Larsson SC, Malik R, Rannikmäe K, Sudlow CL, Dichgans M, Markus HS, Traylor M. Genetic risk, incident stroke, and the benefits of adhering to a healthy lifestyle: cohort study of 306 473 UK Biobank participants. Bmj 2018;363:k4168. doi: 10.1136/bmj.k4168.
13. Benjamin EJ, Blaha MJ, Chiuve SE, Cushman M, Das SR, Deo R, de Ferranti SD, Floyd J, Fornage M, Gillespie C, et al. Heart Disease and Stroke Statistics-2017 Update: A Report From the American Heart Association. Circulation 2017;135(10):e146-e603. doi: 10.1161/cir.0000000000000485.
